# Limited midline myelotomy for visceral pain

**DOI:** 10.3171/2020.6.FOCVID2014

**Published:** 2020-10-01

**Authors:** M. Benjamin Larkin, Robert Y. North, Aditya Vedantam, Ashwin Viswanathan

**Affiliations:** Department of Neurosurgery, Baylor College of Medicine, Houston, Texas

**Keywords:** cancer pain, functional, myelotomy, palliative care, visceral pain

## Abstract

The traditional commissural myelotomy consists of a sagittal cut in the midline and was originally described by Greenfield and performed by Armour in 1926. Today, myelotomy refers to the selective disruption of the ascending visceral pain pathway. The success of the procedure is incumbent on the correct identification of the midline. Limited midline open myelotomy for the treatment of medically intractable abdominal or pelvic visceral cancer pain, with the aid of somatosensory evoked potentials to identify midline, offers patients superior pain relief over similar percutaneous techniques. Multicenter registries are needed to better elucidate the best surgical technique for this procedure.

The video can be found here: https://youtu.be/0unlmwp08po

## Transcript

This video illustrates an open, surgical, limited midline myelotomy for the treatment of visceral malignancy–related pain. The procedure interrupts ascending second-order dorsal column projections that convey visceral pain to the pelvic and lower abdominal organs.^1^

### Rationale for the Procedure

**0:37** The traditional commissural myelotomy consists of a sagittal cut in the midline and was originally described by Greenfield and performed by Armour in 1926.^2^ The goal of this procedure was interruption of both the decussating spinothalamic tract fibers as well as interruption of the dorsal column visceral pain pathway. Today, myelotomy most commonly refers to the selective disruption of the ascending visceral pain pathway and was initially performed by Hirshberg in 1996 via a limited transverse midline transection.^1–3^

**1:14** Information regarding the postsynaptic dorsal column pathways for visceral pain comes from physiological localization and retrograde axon labeling in rodent studies, summarized by Willis and colleagues, among others.^2^

**1:28** The more commonly discussed presynaptic myelinated dorsal column pathways originate in the dorsal root ganglion and project vibration, discriminative touch, and proprioception.

**1:39** In comparison, the postsynaptic unmyelinated dorsal column pathways that originate mainly in the gray matter of lamina 3 through 7 carry visceral pain from the pelvis and lower abdominal organs within the fasciculus gracilis.^2^

**1:55** The majority of projections terminate in the gracile and cuneate nucleus, while some continue further on to the medial and intralaminar nuclei of the thalamus and influence the behavioral and emotional pain responses. There may be further segregation of the dorsal visceral pain projections such that lumbosacral viscera are found within the medial fasciculus gracilis, while thoracic viscera are found more laterally near the dorsal intermediate septum separating the gracilis and cuneatus fasiculi.^2,4,5^

**2:26** The dorsal ascending, visceral pain pathways are distinct from the anterior lateral spinal thalamic pathways that synapse in the superficial lamina of the dorsal horn and carry acute somatic pain and temperature sensation.^2,4–6^

**2:39** Visceral pain is transmitted via the medial lemniscus pathway and is widely divergent owing to its poor localization, in contrast to the precise somatic localization found in the spinothalamic pathway.^2,5,7^

**3:04** While with cordotomy there is risk to surrounding structures, including the reticulospinal and corticospinal tracts, and subsequent respiratory and motor complications, collateral damage from midline myelotomy is usually less clinically severe. Historically attributed symptoms from damage to the dorsal columns and subsequent loss of proprioception was severely debilitating and associated with neurosyphillis.^2,4,8,9^

**3:20** However, from a limited midline myelotomy, there is minimal damage to the dorsal columns and preservation of other sensory pathways. Patients may experience a decrease in proprioception following myelotomy; this is usually not clinically impairing.^5,9,10^

**3:36** This procedure is most commonly utilized in those patients with severe, medically intractable abdominal or pelvic visceral cancer pain, with a life expectancy greater than 3 months and a Karnofsky Performance score of 40 or more.^10^ The role of limited myelotomy for chronic noncancer pain is lacking and outcomes have not been well described.^9^

**3:57 Description of the Setup.** The patient is placed under general endotracheal anesthesia and then into a prone position. Imaging is obtained to confirm the intended thoracic level—typically, T3 or T4 for upper abdominal pain and T6 to T8 for lower abdominal or perineal pain.^9^

### Key Surgical Steps

**4:18** Afterwards, a single-level thoracic wide laminectomy is performed at the intended level to allow visualization of the dorsal root entry zones bilaterally. This helps to identify the midpoint of the spinal cord.

**4:37** A midline dural incision is made, and the dura is opened under tension. Dural retention sutures as well as arachnoid clips are placed. The midline dorsal vein should be preserved by mobilization if it obscures the dorsal median sulcus.

**4:52** The exact midline can be determined by measuring midway between the two route entry zones and the presence of small arterioles penetrating the dorsal median sulcus.

**5:03** The success of the procedure is incumbent on the correct identification and the exact physiological midline. To ensure this, stimulation via a handheld concentric bipolar probe of the dorsal columns is performed while somatosensory evoked potentials are recorded from scalp electrodes. Stable and high-amplitude scalp-derived responses are achieved with 0.2- to 0.3-mA stimulations. The probe is in place generally over the dorsal surface of the spinal cord, and the midline is represented by the inert midline raphe, or rather the area of flat sensory evoked potential responses from the probe, as well as demonstration of phase reversal upon crossing midline.

**5:50** This is best represented here in these electrographic recordings where you can see the change as the probe is placed first at the edge of the dorsal column, then on the dorsal column, then over the midline where the line flattens, and then finally phase reversal is demonstrated upon crossing midline.

**6:08** Once the midline is confirmed, the pia is coagulated via micro bipolar forceps and a 16-gauge angiocatheter is used to create the lesion.

**6:19** The catheter is cut to allow a 5-mm exposed needle tip, which is the desired lesion depth. Four lesions are made in the same location, and each time the needle is rotated 90° in order to create a 0.5 × 5 mm–lesion that extends from the midline bilaterally to the intended depth. It is important to enter the spinal cord entirely perpendicular to the midline in order to minimize the risk of damaging adjacent corticospinal tracts and postoperative motor weakness.^9^

**6:54** Other authors have used a fine forceps or a microdissector to create a similar lesion.^5,10^

**7:00** Meticulous hemostasis is obtained with the use of thrombin-soaked Gelfoam. The dura is closed solely with a running suture. To prevent cerebral spinal fluid leak, it is important to obtain excellent fascial closure, as dural sealants are not used. The patient is typically mobilized on the evening of the surgery.

**7:21** Both the percutaneous mechanical and radiofrequency lesioning offer a minimally invasive option to avoid open surgery and the need for general anesthesia in advanced cancer patients.^9^

**7:34** Preliminary studies suggest that mechanical lesioning, whether open or closed, may be more effective in disrupting the ascending fiber tracts as compared with radiofrequency ablation.^9^

**7:45** The majority of patients have lasting improvements in pain from a minimum of 1 to 2 months to over 30 months. The durability of the procedure is difficult to assess, given the short life expectancy of many patients. Additionally, recurrence of pain is often the result of disease progression.^3,9^

**8:04 Conclusion.** Limited midline open myelotomy for the treatment of visceral malignancy–related pain with the aid of somatosensory evoked potentials to identify midline offers patients who are able to undergo general endotracheal anesthesia and tolerate a prone surgery superior pain relief over similar percutaneous techniques. Multicenter registries are needed to better elucidate the best surgical technique for this procedure. However, for limited midline myelotomy, the open technique remains the preferred first-line approach.

## Author Contributions

Primary surgeon: Viswanathan. Editing and drafting the video and abstract: Larkin, North, Vedantam. Critically revising the work: Larkin, North. Reviewed submitted version of the work: Larkin, North, Vedantam. Approved the final version of the work on behalf of all authors: Larkin.
